# The relationship between patient-reported quality of life and clinician-rated outcome scores in patients with autoimmune encephalitis: a study of the Australian Autoimmune Encephalitis Consortium

**DOI:** 10.1007/s11136-025-04052-4

**Published:** 2025-08-31

**Authors:** Christina Kazzi, Nabil Seery, Sarah Griffith, Robb Wesselingh, Tiffany Rushen, Tracie H. Tan, Katherine Y. Ko, Liora ter Horst, Genevieve Skinner, Laurie McLaughlin, Hannah Ford, Catherine Meade, Marie O’Shea, Katherine Buzzard, Mirasol Forcadela, Andrew Duncan, Anneke van der Walt, Wendyl D’Souza, Udaya Senvieratne, Richard Macdonell, Sudarshini Ramanathan, Stefan Blum, Stephen W. Reddel, Todd A. Hardy, Helmut Butzkueven, Terence J. O’Brien, Rubina Alpitsis, Charles B. Malpas, Mastura Monif

**Affiliations:** 1 Department of Neuroscience, School of Translational Medicine, Monash University, Level 6, Alfred Centre, 99 Commercial Rd, Melbourne, VIC 3004 Australia; 2 Department of Neurology, Alfred Health, Melbourne, VIC Australia; 3 Department of Neurology, Princess Alexandra Hospital, Woolloongabba, QLD Australia; 4 School of Medicine, The University of Queensland, Brisbane, QLD Australia; 5 Department of Neuroscience, Monash Medical Centre, Clayton, VIC Australia; 6 Neuropsychology Unit, Department of Clinical Neurosciences, St Vincent’s Hospital, Melbourne, VIC Australia; 7 Melbourne School of Psychological Sciences, University of Melbourne, Parkville, VIC Australia; 8 Department of Neurology, Austin Health, Melbourne, VIC Australia; 9 Department of Neurosciences, Eastern Health, Melbourne, VIC Australia; 10 Department of Medicine (St Vincent’s Hospital), University of Melbourne, Melbourne, VIC Australia; 11 Department of Neurology, Concord Clinical School, Concord Hospital, Concord, NSW Australia; 12 Translational Neuroimmunology Group, and Kids Neuroscience Centre, Faculty of Medicine and Health, University of Sydney, Camperdown, NSW Australia; 13 Brain and Mind Centre, University of Sydney, Camperdown, NSW Australia; 14 Department of Medicine (Royal Melbourne Hospital), University of Melbourne, Parkville, VIC Australia; 15 Department of Neurology, Royal Melbourne Hospital, Parkville, VIC Australia

**Keywords:** Patient-reported outcomes, Quality of life, Clinical outcome scores, Autoimmune encephalitis

## Abstract

**Background:**

Patients with Autoimmune Encephalitis (AE) commonly report poor quality of life. There is a lack of evidence on whether clinician-rated outcome measures adequately capture patient-reported experiences. This study aimed to characterise long-term quality of life in AE patients and examine its relationship with clinician-rated disability (modified Rankin Score, mRS) and symptom severity (Clinical Assessment Scale in Autoimmune Encephalitis, CASE).

**Methods:**

Patients with AE were recruited through the Australian Autoimmune Encephalitis Consortium Project. Patients with completed NeuroQoL instruments, as well as CASE and/or mRS scores within 6 months of the NeuroQoL were included.

**Results:**

Fifty-four patients with AE (50% female, median age at symptom onset = 49.70 years) completed the NeuroQoL instruments at a median of 50 months (IQR: 18.25–87.50 months) post-disease onset. The median CASE score was 2 (IQR: 0.0–3.0). The median mRS was 1.5 (IQR: 1.0–2.0). In the total AE sample, CASE scores were positively associated with all NeuroQoL domains (*r* = .42−.53), except Fatigue (*r* = .28). Total NeuroQoL, Cognitive Function, Satisfaction in Social Roles and Activities, and Stigma T-scores were significantly higher in seropositive AE patients with an mRS > 2 (*d* = 0.94–1.66). The mRS significantly predicted most NeuroQoL domains in the total AE sample, and the CASE score explained little or no additional variance over and above mRS scores. Similarly, the CASE score was significantly associated with most NeuroQoL domains, with no additional variance explained by mRS scores.

**Discussion:**

The relationships between clinician-rated outcomes and domains of quality of life varied from weak to moderate. This highlights the importance of integrating clinical measures and patient-reported outcomes when assessing outcomes post-AE.

**Supplementary Information:**

The online version contains supplementary material available at 10.1007/s11136-025-04052-4.

## Plain English summary

Patients with autoimmune encephalitis often experience poor quality of life including anxiety, cognitive dysfunction, reduced satisfaction in their social roles and activities, and sleep disturbance. It is unclear whether the outcome scores often used by clinicians accurately reflect the patient’s daily life. This study found that a large proportion of patients reported poor quality of life despite having been categorised as having a ‘good’ functional score based on clinician-rated scores such as the Clinical Assessment Scale in Autoimmune Encephalitis (CASE) and modified Rankin Score (mRS). The CASE and mRS only share weak to moderate relationships with patient-reported quality of life. This highlights the need for clinicians to utilise questionnaires to assess the impact of autoimmune encephalitis on their patients’ functioning, as clinician-rated outcomes do not accurately reflect their experiences. Using valid and reliable measures of a patient’s symptoms is crucial to guide treatment and rehabilitation, increase patient engagement, and enhance long-term outcomes.

## Introduction

Autoimmune encephalitis (AE) is an immune-mediated group of neurological disorders characterised by inflammation in the brain. The subtypes of AE are classified according to neural autoantibodies that target intracellular or cell-surface antigens [1]. When an associated antibody is not identifiable, the subtype is classified as ‘seronegative’ [2]. Patients typically present with neuropsychiatric symptoms, including behavioural change, hallucinations, cognitive deficits, movement abnormalities, dysautonomia, and seizures [3, 4].

Patients are significantly affected by these symptoms and report poor quality of life [5–8]but this is not accurately captured by the most commonly used outcome metric, the modified Rankin scale (mRS) [5, 9]. The mRS—a scale originally developed to measure disability after stroke—is considered to be imperfect in assessing outcomes following AE as it neglects symptoms such as seizures, cognitive deficits, and psychopathology, which impact on quality of life [5, 6, 10]. Cohort studies of leucine-rich glioma-inactivated 1 (LGI1) AE and N-methyl-D-aspartate receptor (NMDAR) AE have demonstrated poor correlation between mRS and impairments such as cognition, fatigue, and mood [7, 11] As an illustrative example, de Bruijn et al. [12] studied a sample of paediatric patients with anti-NMDAR encephalitis, and found deficits in attention, verbal memory, language, and executive function, as well as poor quality of life and significant fatigue despite good disease outcome, as measured by the mRS or Paediatric Cerebral Performance Category scale. Likewise, 60% of patients with anti-Contactin-associated Protein-Like 2 (CASPR2) encephalitis were found to have persistent fatigue and neuropathic pain at 6 months and 48 months post-treatment [13]. Furthermore, patients with good mRS scores (mRS 0–2) had variable quality of life scores, highlighting that clinical outcome scores do not accurately reflect symptom burden in patients [13].

To address this need for AE-specific outcome measures, clinicians and researchers have developed the Clinical Assessment Scale in Autoimmune Encephalitis (CASE) [14]. The CASE score is comprised of nine items: seizures, memory dysfunction, language function, psychiatric symptoms, consciousness, dyskinesia/dystonia, gait and ataxia, limb weakness, and brainstem dysfunction [14]. While the CASE score better assesses the clinical symptoms of AE, studies have yet to investigate whether it also reflects patient-reported health-related quality of life.

While the importance of patients’ lived experiences is increasingly recognised, a recent systematic review concluded that the use of health-related quality of life measures in AE literature remains sparse [15]. The review also suggested that future research utilise the Quality of Life in Neurological Disorders scales (NeuroQoL), as it has been validated in several neurological conditions [16] and covers several domains of quality of life, as opposed to measures such as the 36-item Short Form 36 Health Survey. In the broader encephalitis literature (infectious, as well as autoimmune), a 2022 systematic review identified 37 outcome measures used in encephalitis [17]. Amongst the most frequently used outcome measures were the Euro-QoL-5D and the modified Rankin Scale [17]. The authors concluded that the Liverpool Outcome Score [18], a tool that combines both clinician and patient reports, was the most appropriate as it was the only tool validated in the encephalitis population at the time of the review [17]. Similarly, Brenner et al. [19] also identified that clinical outcome measures in patients with encephalitis have yet to be validated in detecting neurocognitive, functional, and health status. Since then, the LGI1-ANTibody encephalitis outcome RatiNg scale (LANTERN) has been developed [20], although other autoimmune encephalitides remain without a validated outcome score.

In summary, while outcome measures such as the mRS and CASE are commonly used in research and clinical practice, these tools have been purported to lack the ability to capture patient-reported outcomes such as sleep, fatigue, mood, and psychosocial well-being. Research is needed to explicitly identify and bring awareness to these AE impacts on day-to-day life, so that clinicians do not overlook burdensome symptoms by purely relying on these traditional outcome measures. Measuring patient-reported outcomes and their association with clinician-derived conventional outcomes measures, can provide comprehensive and patient-centred understanding of AE disease burden.

The primary aim of this study was to characterise the long-term quality of life of patients with AE, and investigate the relationship between quality of life and clinical scores of symptom severity (CASE) and functional disability (mRS). More specifically, the study aimed to: (1) compare quality of life between patients with AE and the normative sample, as well as between patients with seropositive AE and antibody negative AE; (2) assess the frequency and severity of poor quality of life in the AE population; (3) explore associations between demographic and treatment variables with quality of life; (4) examine the relationship between quality of life and the CASE score; (5) examine the relationship between quality of life and the mRS; and (6) determine whether the CASE score explained quality of life over the mRS, and vise versa.

## Methods

### Participants

In this multi-centre mixed (retrospective and prospective) cohort study, participants were recruited via the Australian Autoimmune Encephalitis Consortium through ten Australian hospitals: Alfred Health, Melbourne Health, Eastern Health, Monash Health, Austin Health, St Vincent’s Health, Barwon Health, Peninsula Health, Concord Repatriation General Hospital, and Princess Alexandra Hospital. Patients with AE were identified retrospectively through hospital medical records using the International Classification of Disease (ICD)-10 codes (G048, G258, G608, G049 and M359) between January 2008 and April 2024, or prospectively in the inpatient and ambulatory setting. Inclusion criteria were a diagnosis of ‘possible’ AE, ‘autoantibody-negative but probable AE’, ‘definite autoimmune limbic encephalitis’, or ‘anti-N-methyl-D-aspartate receptor encephalitis’ as defined by Graus et al. [21] or a diagnosis of other antibody-mediated encephalitis based on clinical features and positive serum or cerebrospinal fluid autoantibodies. All patients provided informed consent, and the study was approved by the central Human Research Ethics Committee at Alfred Health (HREC/17/Alfred/168).

### Measures

Medical records were reviewed for patient demographics, pre-existing medical history, antibody profile, clinical features from the time of symptom onset, paraclinical findings (i.e., full blood examination, cerebrospinal fluid (CSF), electroencephalogram (EEG), neuroimaging findings), and treatment.

Initial admission, discharge, twelve-month, and final follow-up CASE and mRS scores were either (1) retrospectively assigned by neurologists (N.S., R.W.) through medical record review or (2) prospectively by the patient’s treating clinician during review in outpatient clinic. The mRS and CASE score graded closest to the completion of the quality of life measure (i.e., NeuroQoL) was used, if the time difference between measures was less than six months.

Participants were invited to complete seven NeuroQoL scales: Anxiety (Short Form v1.0), Fatigue (Short Form v1.0), Sleep Disturbance (Short Form v1.0), Positive Affect and Wellbeing (Short Form v1.0), Satisfaction with Social Roles and Activities (Short Form v1.1), Cognitive Function (v2.0), and Stigma (Short Form, v1.0). Participants were sent the NeuroQoL to complete online via REDCAP [22, 23] or a printed copy through the mail. The raw scores of the NeuroQoL scales were converted into T-scores (mean = 50, standard deviation = 10) using the HealthMeasures Scoring Service (https://www.assessmentcenter.net/ac_scoringservice) and relevant reference populations, including clinical reference populations and the United States general population (https://www.healthmeasures.net/score-and-interpret/interpret-scores/neuro-qol/reference-populations). T-scores were reversed for Cognitive Function, Positive Affect and Wellbeing, and Satisfaction with Social Roles and Activities so that that higher scores indicated worse quality of life for all NeuroQoL scales. The mean of the seven NeuroQoL scaled scores was used to calculate a ‘total NeuroQoL’ score for each patient. As per the NeuroQoL score cut-off points (https://www.healthmeasures.net/score-and-interpret/interpret-scores/neuro-qol/neuro-qol-score-cut-points), T-scores less than 55 were classified as “normal”, 55–60 “mild”, 61–70 “moderate”, and greater than 70 “severe”.

Seventy-four patients in the Australian Autoimmune Encephalitis Consortium Project completed the NeuroQoL at least once between February 2021 and April 2024. Of these 74 patients, 54 patients also had a mRS and/or CASE score completed 6 months prior to or following their completion of the NeuroQoL and were included in the study. That is, mRS and/or CASE scores were collected within a 6-month window either before or after the patient completed the NeuroQoL assessment.

### Statistical analyses

Statistical analyses were performed using JASP (0.19.2) and figures were created with GraphPad Prism (10.4.1). The following analyses were used to examine the relationship between demographic, treatment, and disease variables on NeuroQoL scores. Student’s single sample t-tests were used to investigate whether mean NeuroQoL T-scores differed from the normative mean. Welch’s t-test was used to test whether there were differences between (1) males and females, (2) the seropositive and seronegative group, (3) participants with drug resistant epilepsy (DRE) at final follow up and those without, (4) participants who received first line treatment only and those who received second line treatment, and (5) participants with pre-existing psychiatric comorbidities and those without. Following Delacre et al. [24], Welch’s t-tests were run throughout this study rather than Student’s t-test to reduce type II error. Correlations were used to investigate the association between age at symptom onset, time since symptom onset, and time to treatment with NeuroQoL scores. As results were comparable between Pearson’s and Spearman’s correlations, Pearson’s product moment correlation coefficients were reported.

The following analyses were conducted for the seropositive AE cases, seronegative AE cases, and the combined (total) AE cohort to examine the relationship between NeuroQoL scores and clinical outcome scores–the CASE and mRS. Correlations were computed to investigate the relationship between NeuroQoL scores and the CASE score. As results were similar for parametric and non-parametric tests, Pearson’s product moment correlation coefficients were reported. Welch’s independent samples t-tests were used to compare mean NeuroQoL scores between dichotomized mRS scores. The mRS scores were dichotomized in two ways: (1) using the conventional cut-off of mRS ≤ 2 for favourable outcome, and (2) using a cut-off of mRS ≤ 1 to define “true” independence, where symptoms have no impact on a patient’s ability to perform their usual duties and activities.

Finally, hierarchical linear regression analyses were used to assess the contribution of mRS and CASE score in explaining quality of life (NeuroQoL) metrics in the total AE sample. The clinical outcome scores were entered in a hierarchical manner. We modelled this twice: (1) mRS was entered in the first step, and CASE was entered in the second step, and (2) CASE was entered in the first step, and mRS was entered in the second step.

To address the number of statistical tests conducted in this study, a False Discovery Rate (FDR) correction was applied. Findings that remained significant after FDR correction are marked with †. A list of all corrected p-values is provided in Supplementary Table 1.

## Results

Fifty-four patients with AE were included in this study. Twenty-seven (50%) were female. The median age at symptom onset was 49.70 years (IQR: 27.36–65.37). Subtypes included anti-LGI encephalitis (*n* = 15, 28%), anti-NMDAR encephalitis (*n* = 15, 28%), anti-GFAP encephalitis (*n* = 1, 2%), possible antibody-negative autoimmune encephalitis (*n* = 12, 22%), probable seronegative autoimmune encephalitis (*n* = 3, 6%), seronegative limbic encephalitis (*n* = 6, 11%), and encephalitis associated with onconeuronal antibodies (*n* = 2, 4%, anti-Hu and anti-Yo). Of the 53 (98%) patients that were treated, 41 (77%) received pulse steroids, 39 (68%) received steroids, 47 (89%) received IVIg, ten received plasma exchange (19%), 31 (58%) received rituximab, six (11%) received cyclophosphamide, one (2%) received bortezomib, 13 (25%) received mycophenolate, three (6%) received methotrexate, and ten (19%) received azathioprine. The median time from symptom onset to treatment was 40 days (IQR: 20–174 days). The median time from symptom onset to completion of the NeuroQoL survey was 50 months (IQR: 18.25–87.50). Eight (15%) had drug-resistant epilepsy (failure of adequate trials of two tolerated and appropriately chosen and used AED schedules to achieve seizure freedom) at final follow-up.

### Clinical outcome measures

Of the 54 participants, 49 had all three outcome measures—NeuroQoL, CASE, and mRS—completed with the 6-month time-matching criterion. Five did not have a CASE score time-matched with the NeuroQoL.

Fifty-four participants had time-matched NeuroQoL and mRS scores. mRS scores were completed a median of three days prior to the NeuroQoL (IQR: -36.75–15.75). Median mRS was 1.5 (IQR: 1.0–2.0). Of the 54 participants, 12 (22%) had an mRS of 0, 15 (28%) had an mRS of 1, 14 (26%) had an mRS of 2, 11 (20%) had an mRS of 3, one (2%) had an mRS of 4, and one (2%) had an mRS of 5.

Forty-nine participants had time-matched NeuroQoL and CASE scores. CASE scores were completed a median of 0 days from NeuroQoL completion (IQR: -37.00–21.00). The median CASE score was 2 (IQR: 0–3).

#### Variables associated with quality of life

There were no significant correlations between NeuroQoL scores and age at symptom onset, time since symptom onset, and time to treatment in the total AE sample (Supplementary Table 2).

Welch’s t-tests found no significant difference in NeuroQoL scores between females and males, and between patients who received second line treatment (i.e., rituximab and/or cyclophosphamide) and those who only received first-line treatment (Supplemental Table 3). Premorbid anxiety and/or depression was associated with significantly worse Total NeuroQoL (*t*(20.64) = -2.33, *p* = .030, *d* = -0.74), Anxiety (*t*(21.67) = -2.67, *p* = .014, *d* = -0.84), Cognitive Function (*t*(28.04) = -2.19, *p* = .037, *d* = -0.64), Positive Affect and Wellbeing (*t*(26.76) = -2.65, *p* = .013†, *d* = -0.79), and Satisfaction with Social Roles and Activities (*t*(28.59) = -2.94, *p* = .006^†^, *d* = -0.86). Fatigue, Sleep Disturbance, and Stigma did not significant differ between participants with and without premorbid anxiety and/or depression (Supplemental Table 3).

Welch’s t-tests demonstrated that patients with drug-resistant epilepsy at final follow up had worse Total NeuroQoL (*t*(10.83) = -3.09, *p* = .011†, *d* = -1.11), Anxiety (*t*(12.11) = -3.76, *p* = .003^†^, *d* = -1.28), Cognitive Function (*t*(27.68) = -5.58, *p* < .001†, *d* = -1.47), Satisfaction in Social Roles and Activities (*t*(13.47) = -2.69, *p* = .018, *d* = -0.87), Sleep (*t*(9.29) = -2.66, *p* = .025, *d* = -1.04), and Stigma (*t*(15.164) = -3.80, *p* = .002^†^, *d* = -1.18) compared to patient without drug-resistant epilepsy. There was no significant difference for Fatigue (*t*(9.58) = -0.98, *p* = .350, *d* = -0.38) and Positive Affect and Wellbeing (*t*(8.52) = -0.49, *p* = .636, *d* = -0.20) (Supplementary Table 3).

### Quality of life in AE

#### Does quality of life differ between patients with AE and the normative sample?

**Fig. 1:**
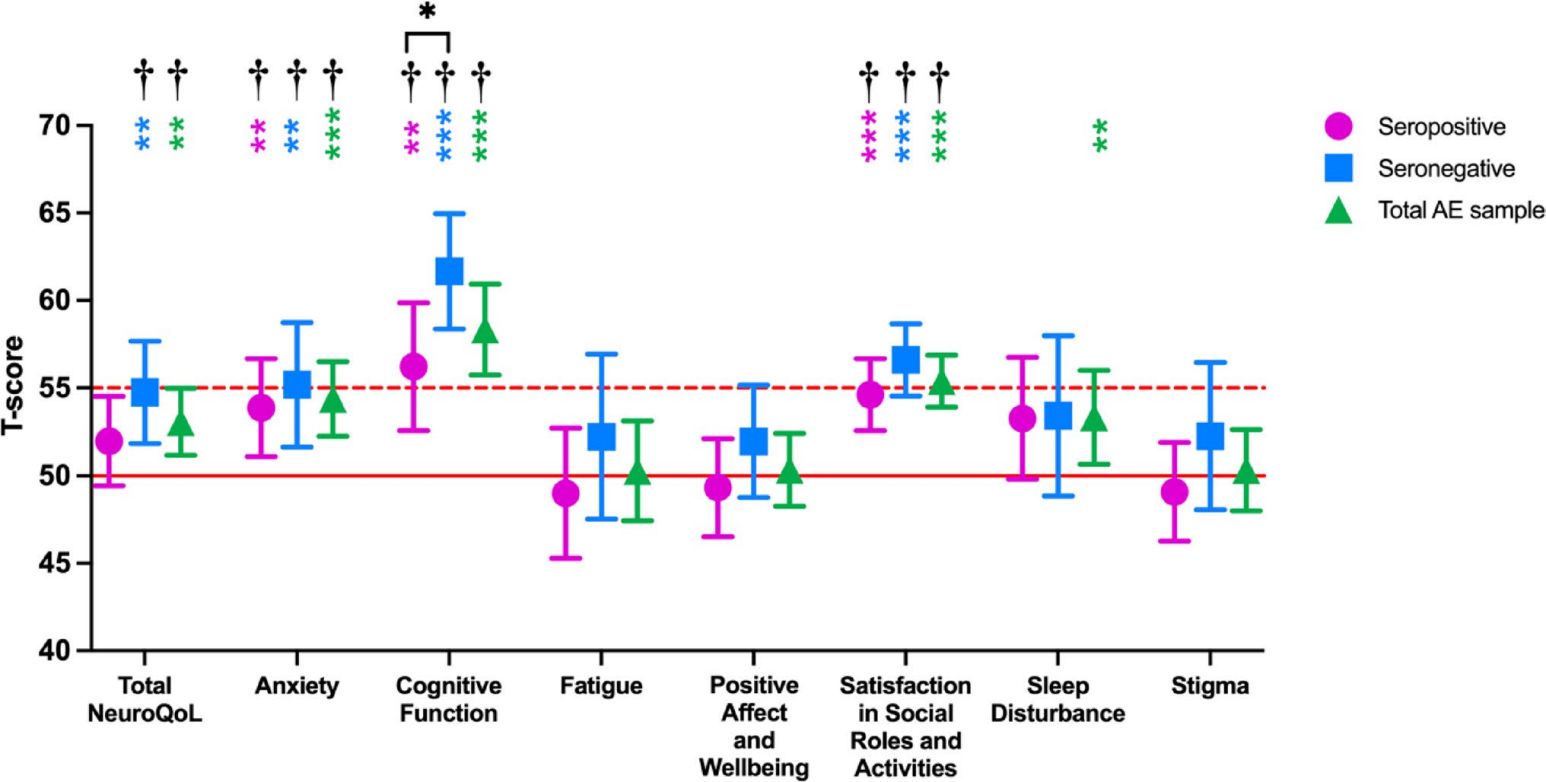
Means and 95% CI of NeuroQoL domain T-scores for the seropositive cases only, seronegative cases only, and the total AE sample. A solid red reference line is drawn at the normative mean of 50 T-score. A dotted red reference line is drawn at 55 T-score; T-scores above this reference line are considered abnormal. Coloured asterisks indicate whether the mean NeuroQoL T-score for the indicated group is significantly different from the normative mean. Black asterisks with connecting bases indicate that the mean NeuroQoL T-score differs significantly between the seropositive and seronegative samples; however this difference did not survive False Discovery Rate (FDR) correction. *p < .05, **p < .01, ***p < .001, ^†^survives FDR correction

Student’s single sample t-tests revealed significantly higher mean T-scores for total NeuroQoL, Anxiety, Cognitive Function, Satisfaction in Social Roles and Activities, and Sleep Disturbance in the total AE sample compared to the normative sample (Table 1; Fig. 1). When the seropositive and seronegative AE groups were analysed separately, both groups demonstrated elevated T-scores for Anxiety, Cognitive Function, and Satisfaction in Social Roles and Activities, while only the seronegative group had significantly elevated T-scores for Total NeuroQoL compared to the normative sample. Not all significant differences survived FDR correction (See Supplementary Table 1).

**Table 1:** Mean Neuroqol T-Scores in the seropositive cases only, seronegative cases only, and total AE sample compared with the normative population

Scale	Seropositive AE only					Seronegative AE only					Total AE sample (seropositive + seronegative cases)				
	Mean (SD)	t	df	*p*	*d*	Mean (SD)	t	df	*p*	*d*	Mean (SD)	t	df	*p*	*d*
Total NeuroQoL	51.97 (7.07)	1.58	31	0.125	0.28	54.76 (6.40)	3.41	20	0.003^†^	0.74	53.08 (6.89)	3.25	52	0.002^†^	0.45
Anxiety	53.88 (7.86)	2.84	32	0.008^†^	0.49	55.19 (7.79)	3.05	20	0.006^†^	0.67	54.39 (7.78)	4.14	53	< 0.001^†^	0.56
Cognitive Function	56.22 (10.29)	3.48	32	0.001^†^	0.61	61.67 (7.25)	7.38	20	< 0.001^†^	1.61	58.34 (9.53)	6.43	53	< 0.001^†^	0.88
Fatigue	49.01 (10.34)	-0.54	31	0.591	-0.10	52.23 (10.34)	0.99	20	0.334	0.22	50.29 (10.36)	0.20	52	0.842	0.03
Positive Affect and Wellbeing	49.33 (7.91)	-0.49	32	0.628	-0.09	51.96 (7.01)	1.28	20	0.214	-0.28	50.35 (7.61)	0.34	53	0.736	0.05
Satisfaction with Social Roles and Activities	54.63 (5.78)	4.59	32	< 0.001^†^	0.80	56.61 (4.53)	6.68	20	< 0.001^†^	1.46	55.40 (5.38)	7.38	53	< 0.001^†^	1.00
Sleep Disturbance	53.29 (9.80)	1.93	32	0.063	0.34	53.42 (10.04)	1.56	20	0.134	0.34	53.34 (9.80)	2.50	53	0.015	0.34
Stigma	49.09 (7.97)	-0.66	32	0.515	-0.11	52.26 (9.21)	1.13	20	0.274	0.25	50.32 (8.53)	0.28	53	0.782	0.04

Scores are T-scores (M = 50, SD = 10). T-scores between 55 and 60 are classified as “mild”, 61 to 70 are classified as “moderate”, and greater than 70 are “severe”

^†^Survives FDR correction

#### Does quality of life differ between patients with seropositive AE and seronegative AE?

Welch’s t-tests demonstrated that the seronegative AE sample had significantly higher Cognitive Function T-scores (*M* = 61.67, *SD* = 7.25*)* than the seropositive AE sample (*M* = 56.22, *SD* = 10.29, *t*(51.37) = 2.28, *p* = .027, *d* = 0.61; Fig. 1, Supplementary Table 4). However, this significant difference did not survive FDR correction. There were no significant differences in T-score for the other NeuroQoL domains.

#### Frequency and severity of poor quality of life

**Fig. 2:**
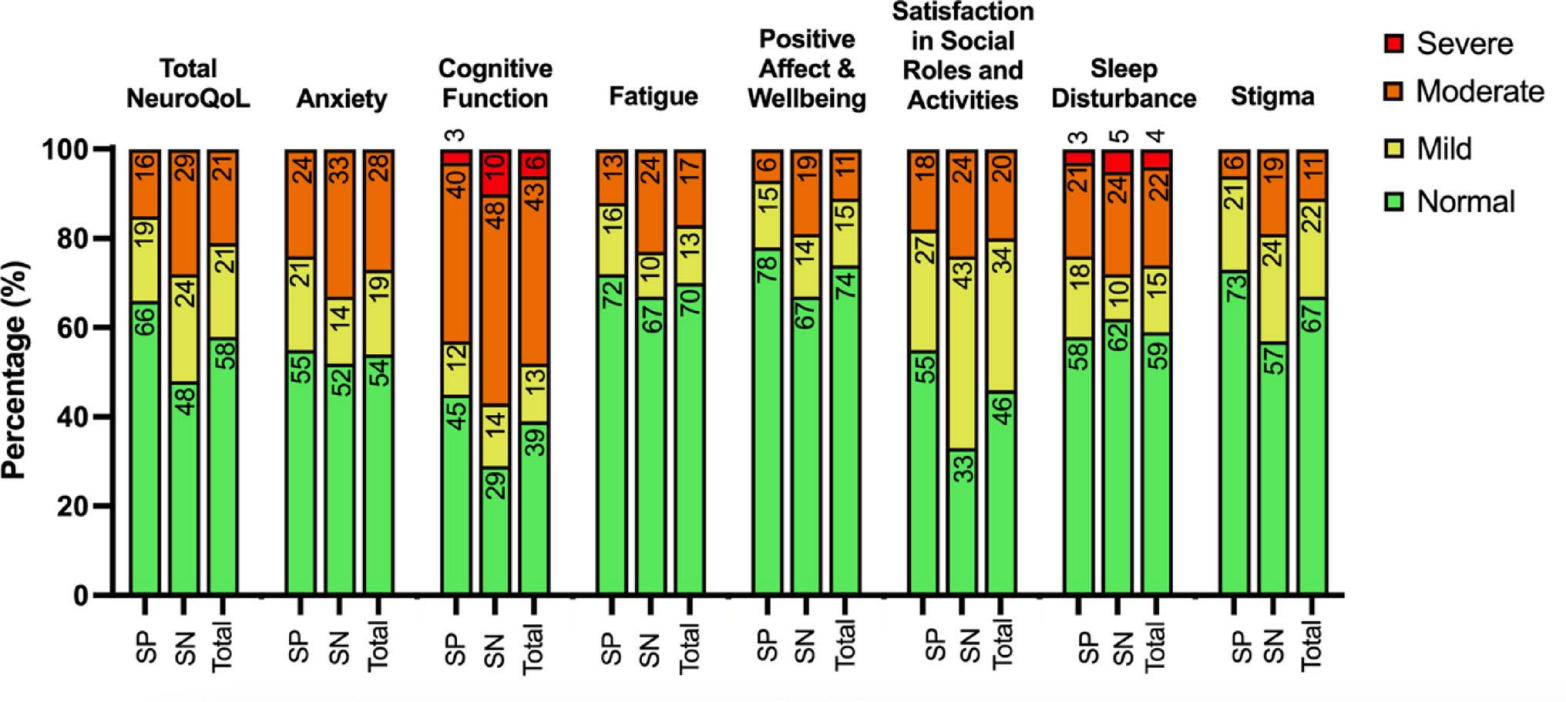
Illustrates the percentage of the AE sample reporting normal and poor quality of life. Poor quality of life is categorised based on the severity of symptoms; mild symptoms are coloured in yellow, moderate symptoms are coloured in orange, and severe symptoms are coloured in red. T-scores less than 55 are classified as “normal”, 55–60 “mild”, 61–70 “moderate”, and greater than 70 “severe”. SP, seropositive; SN, seronegative; Total; Total AE sample

NeuroQoL T-scores are categorised based on symptom severity: normal, mild, moderate, and severe. Figure 2 and Supplementary Table 5 illustrate that a large proportion of participants with AE endorse poor quality life (i.e., mild to severe symptoms) across the surveyed domains.

### Relationship between Neuroqol scores and CASE score

In the total AE sample, the CASE score significantly correlated with Total NeuroQoL, Anxiety, Cognitive Function, Positive Affect and Wellbeing, Satisfaction in Social Roles and Activities, Sleep Disturbance, and Stigma, but not Fatigue (Table 2). This is similar to the seropositive AE group, which also demonstrated significant correlations with the CASE score and the same NeuroQoL domains, with the exception of Sleep Disturbance. In the seronegative AE sample only Total NeuroQoL, Anxiety, Positive Affect and Wellbeing, and Sleep Disturbance were significantly correlated with the CASE scores. Not all these correlations survived FDR correction (Supplementary Table 1).

**Table 2:** Pearson’s correlations between neuroqol domain T-scores and the CASE score

NeuroQoL domain	Seropositive AE	Seronegative AE	Total AE sample (seropositive + seronegative)
Total NeuroQoL	0.533** ^†^	0.521*	0.513*** ^†^
Anxiety	0.417*	0.458*	0.431** ^†^
Cognitive Function	0.659*** ^†^	0.380	0.494*** ^†^
Fatigue	0.258	0.290	0.276
Positive Affect andWwellbeing	0.382*	0.506*	0.437** ^†^
Satisfaction with Social Roles and Activities	0.574*** ^†^	0.336	0.427** ^†^
Sleep Disturbance	0.335	0.525*	0.420** ^†^
Stigma	0.676*** ^†^	0.433	0.532*** ^†^

*p < .05, **p < .01, ***p < .001, ^†^survives FDR correction

### Relationship between Neuroqol scores and mRS

**Fig. 3:**
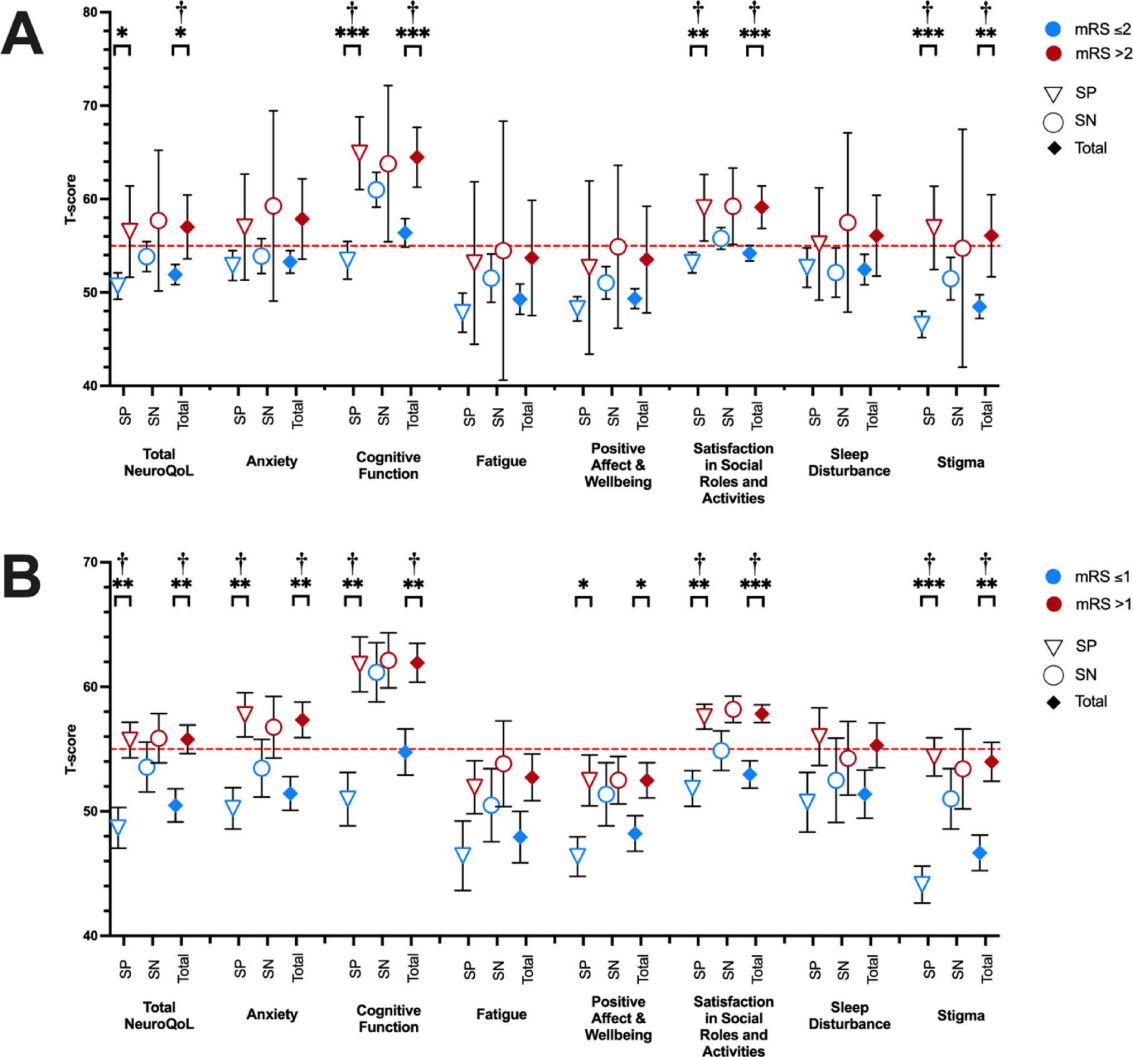
**A**, NeuroQoL scores (mean and 95% CI) stratified by conventional dichotomised mRS outcome (Good, mRS ≤ 2; Poor, mRS of > 2). **B**, NeuroQoL T-scores (mean and 95% CI) stratified by dichotomised mRS outcome based on ability to perform usual duties and activities (can perform usual activities, mRS ≤; unable to perform usual duties and activities, mRS of > 1). A dotted red reference line is drawn at 55 T-score; T-scores above this reference line are considered abnormal. *SP*, seropositive; *SN*, seronegative; *Total*; Total AE sample. *p < .05, **p < .01, ***p < .001, ^†^survives FDR correction

Table 3; Fig. 3 illustrate the distribution of NeuroQoL T-scores stratified by mRS outcome, which was dichotomised in two ways. First, using the conventional mRS score for favourable outcome (mRS ≤ 2), and second, using a stricter definition of independence whereby a patient’s symptoms does not interfere with their ability to perform their usual duties and activities (mRS ≤ 1).

**Table 3:** NeuroQoL T-scores stratified by dichotomised mRS

NeuroQoL domain	mRS		t	df	*p*	*d*	mRS		t	df	*p*	*d*
	≤ 2	> 2					≤ 1	> 1				
	Mean (SD)	Mean (SD)					Mean (SD)	Mean (SD)				
Seropositive AE cases												
Total	50.70 (7.06)	56.53 (5.29)	2.38	12.70	0.033	0.94	48.67 (6.74)	55.72 (5.53)	3.25	29.86	0.003^†^	1.14
Anxiety	52.88 (8.04)	57.00 (6.79)	1.43	13.87	0.176	0.55	50.24 (6.90)	57.75 (7.12)	3.08	30.69	0.004^†^	1.08
Cognitive Function	53.44 (10.08)	64.91 (4.67)	4.40	26.35	< 0.001^†^	1.46	50.98 (8.84)	61.79 (8.84)	3.51	30.88	0.001^†^	1.22
Fatigue	47.84 (10.46)	53.16 (9.41)	1.29	10.56	0.225	0.53	46.43 (11.51)	51.93 (8.25)	1.57	28.86	0.128	0.55
Positive Affect and Wellbeing	48.26 (6.53)	52.68 (11.09)	1.07	8.61	0.314	0.49	46.37 (6.58)	52.48 (8.17)	2.36	28.82	0.025	0.82
Satisfaction with Social Roles and Activities	53.20 (5.54)	59.08 (4.26)	3.14	15.33	0.007^†^	1.19	51.86 (5.92)	57.59 (3.98)	3.30	28.11	0.003^†^	1.14
Sleep Disturbance	52.67 (10.56)	55.20 (7.18)	0.77	17.58	0.454	0.28	50.73 (9.88)	56.00 (9.25)	1.58	31.00	0.124	0.55
Stigma	46.58 (7.93)	56.93 (5.32)	4.41	15.58	< 0.001^†^	1.66	44.12 (6.12)	54.37 (6.15)	4.80	30.86	< 0.001^†^	1.67
Seronegative AE cases												
Total	53.84 (6.41)	57.71 (6.07)	1.23	7.04	0.260	0.62	53.55 (6.34)	55.87 (6.56)	0.82	18.92	0.421	0.36
Anxiety	53.91 (7.46)	59.28 (8.20)	1.31	6.22	0.238	0.69	53.46 (7.33)	56.76 (8.21)	0.97	19.00	0.343	0.42
Cognitive Function	61.00 (7.48)	63.80 (6.74)	0.79	7.38	0.455	0.39	61.17 (7.52)	62.19 (7.33)	0.29	18.70	0.773	0.13
Fatigue	51.53 (10.34)	54.48 (11.17)	0.52	6.31	0.618	0.27	50.49 (9.29)	53.82 (11.41)	0.74	18.80	0.471	0.32
Positive Affect and Wellbeing	51.04 (6.97)	54.90 (7.02)	1.07	6.68	0.320	0.55	51.37 (8.00)	52.50 (6.323)	0.36	17.14	0.726	0.16
Satisfaction with Social roles and Activities	55.79 (4.64)	59.24 (3.285)	1.85	9.55	0.096	0.86	54.87 (5.04)	58.19 (3.53)	1.73	15.96	0.102	0.76
Sleep Disturbance	52.14 (10.54)	57.50 (7.73)	1.23	9.17	0.249	0.58	52.49 (10.72)	54.26 (9.82)	0.39	18.35	0.698	0.17
Stigma	51.48 (9.08)	54.74 (10.26)	0.64	6.10	0.548	0.34	51.00 (7.68)	53.41 (10.66)	0.60	18.12	0.557	0.26
Total AE sample (seropositive + seronegative cases)												
Total	51.92 (6.91)	57.02 (5.39)	2.69	22.69	0.013^†^	0.82	50.48 (6.90)	55.78 (5.86)	3.02	50.24	0.004^†^	0.83
Anxiety	53.28 (7.74)	57.88 (7.12)	1.99	21.77	0.060	0.62	51.43 (7.07)	57.34 (7.44)	2.99	51.86	0.004^†^	0.82
Cognitive Function	56.39 (9.79)	64.49 (5.31)	3.81	38.43	< 0.001^†^	1.03	54.76 (9.63)	61.93 (8.11)	2.96	50.54	0.005^†^	0.81
Fatigue	49.28 (10.45)	53.71 (9.70)	1.37	19.12	0.188	0.44	47.93 (10.74)	52.73 (9.54)	1.72	50.68	0.092	0.47
Positive Affect and Wellbeing	49.34 (6.76)	53.53 (9.45)	1.48	16.08	0.158	0.51	48.22 (7.41)	52.49 (7.34)	2.13	52.00	0.038	0.58
Satisfaction with Social Roles and Activities	54.21 (5.30)	59.14 (3.76)	3.70	28.46	< 0.001^†^	1.07	52.96 (5.71)	57.84 (3.74)	3.71	44.85	< 0.001^†^	1.01
Sleep Disturbance	52.47 (10.42)	56.09 (7.17)	1.41	29.51	0.169	0.41	51.38 (10.03)	55.29 (9.34)	1.48	51.74	0.144	0.40
Stigma	48.50 (8.15)	56.09 (7.27)	3.18	22.41	0.004^†^	0.98	46.67 (7.41)	53.98 (8.11)	3.46	51.59	0.001^†^	0.94

^†^Survives FDR correction

#### Stratified by mRS ≤ 2 and mRS > 2

In the total AE sample, Welch’s t-tests demonstrated that participants with mRS scores > 2 had significantly higher total NeuroQoL, Cognitive Function, Satisfaction with Social Roles and Activities, and Stigma T-scores than patients with mRS ≤ 2 (Fig. 3; Table 3). There was no significant difference between mRS groups for Anxiety, Fatigue, Positive Affect and Wellbeing, and Sleep Disturbance. The same pattern of significant differences between mRS groups across the NeuroQoL domains was also found for the seropositive AE cases, but not the seronegative AE cases. There were no significant differences in NeuroQoL T-scores between mRS ≤ 2 and mRS > 2 in the seronegative AE cases. Not all these comparisons survived FDR correction (Supplementary Table 1).

#### Stratified by mRS ≤ 1 and mRS > 1

In the total AE sample, Welch’s t-tests demonstrated that participants with mRS scores > 1 had significantly higher total NeuroQoL, Anxiety, Cognitive Function, Positive Affect and Wellbeing, Satisfaction with Social Roles and Activities, and Stigma than patients with mRS ≤ 1 (Fig. 3; Table 3). Most of these comparisons survived FDR correction (Supplementary Table 1). There was no significant difference between mRS groups for Fatigue or Sleep Disturbance. The same pattern of differences between mRS groups for the NeuroQoL domain T-scores was also found for the seropositive AE cases, but not the seronegative AE cases. There were no significant differences in NeuroQoL T-scores between mRS ≤ 1 and mRS > 1 in the seronegative AE cases.

### Does the CASE score explain quality of life over the mRS?

For the total AE sample, hierarchical linear regression analysis identified that the mRS explained a significant proportion of variance for Total NeuroQoL (R² = 0.21, *p* = .001) but the addition of the CASE score did not significantly improve the model (ΔR² = 0.04, *p* = .130). A similar pattern was observed in most NeuroQoL domains. Between 11 and 28% of the variance for Anxiety, Cognitive Function, Positive Affect and Wellbeing, Satisfaction with Social Roles and Activities, and Stigma was explained by mRS, with additional variance explained by the CASE score for Positive Affect and Wellbeing only (Table 4). The mRS did not significantly explain Fatigue and Sleep Disturbance, however, the CASE score did account for an additional 12% of the variance of Sleep Disturbance (*p* = .011).

**Table 4:** Results of the hierarchical linear regression assessing the contribution of mRS and CASE in explaining Neuroqol scores

Do CASE scores explain NeuroQoL scores over mRS?															
NeuroQoL domain	Seropositive AE					Seronegative AE					Total AE sample (seropositive + seronegative)				
	mRS		mRS + CASE			mRS		mRS + CASE			mRS		mRS + CASE		
	R^2^	*p*	R^2^	Δ R^2^	*p*	R^2^	*p*	R^2^	Δ R^2^	*p*	R^2^	*p*	R^2^	Δ R^2^	*p*
Total NeuroQoL	0.24	0.008^†^	0.28	0.04	0.237	0.12	0.143	0.29	0.17	0.060	0.21	0.001^†^	0.27	0.06	0.056
Anxiety	0.14	0.044	0.17	0.03	0.320	0.22	0.036	0.24	0.02	0.518	0.18	0.003^†^	0.20	0.01	0.233
Cognitive Function	0.38	< 0.001^†^	0.44	0.05	0.143	0.08	0.225	0.145	0.06	0.275	0.28	< 0.001^†^	0.29	0.02	0.330
Fatigue	0.06	0.204	0.07	0.01	0.691	0.02	0.609	0.12	0.10	0.184	0.05	0.142	0.08	0.03	0.232
Positive Affect and Wellbeing	0.16	0.034	0.16	0.00	0.810	0.04	0.413	0.37	0.33	0.008^†^	0.11	0.020	0.19	0.08	0.042
Satisfaction with Social Roles and Activities	0.30	0.002^†^	0.33	0.04	0.225	0.12	0.134	0.13	0.01	0.662	0.24	< 0.001^†^	0.24	0.00	0.674
Sleep Disturbance	0.05	0.231	0.16	0.10	0.086	0.10	0.177	0.30	0.20	0.040	0.07	0.070	0.19	0.12	0.011^†^
Stigma	0.50	< 0.001^†^	0.50	0.00	0.652	0.07	0.279	0.21	0.14	0.097	0.28	< 0.001^†^	0.31	0.03	0.137

Do mRS scores explain NeuroQoL scores over CASE?															
NeuroQoL domain	Seropositive AE					Seronegative AE					Total AE sample (seropositive + seronegative)				
	CASE		CASE + mRS			CASE		CASE + mRS			CASE		CASE + mRS		
	R^2^	*p*	R^2^	Δ R^2^	*p*	R^2^	*p*	R^2^	Δ R^2^	*p*	R^2^	*p*	R^2^	Δ R^2^	*p*
Total NeuroQoL	0.28	0.003^†^	0.28	0.00	0.960	0.27	0.018	0.29	0.01	0.567	0.26	< 0.001^†^	0.27	0.01	0.568
Anxiety	0.17	0.025	0.17	0.00	0.896	0.21	0.042	0.24	0.03	0.413	0.19	0.002^†^	0.20	0.02	0.351
Cognitive Function	0.43	< 0.001^†^	0.44	0.00	0.834	0.14	0.099	0.15	0.00	0.906	0.24	< 0.001^†^	0.29	0.05	0.086
Fatigue	0.07	0.185	0.07	0.00	0.888	0.08	0.216	0.12	0.03	0.451	0.08	0.058	0.08	0.00	0.949
Positive Affect and Wellbeing	0.15	0.041	0.16	0.01	0.545	0.26	0.023	0.37	0.12	0.097	0.19	0.002^†^	0.19	0.00	0.887
Satisfaction with Social Roles and Activities	0.33	0.001^†^	0.33	0.00	0.831	0.11	0.147	0.13	0.02	0.567	0.18	0.002^†^	0.25	0.06	0.054
Sleep Disturbance	0.11	0.076	0.16	0.04	0.256	0.28	0.018	0.30	0.03	0.426	0.18	0.003^†^	0.19	0.02	0.352
Stigma	0.46	< 0.001^†^	0.50	0.04	0.141	0.19	0.057	0.21	0.02	0.511	0.28	< 0.001^†^	0.31	0.03	0.178

*CASE* Clinical Assessment Scale in Autoimmune Encephalitis, *mRS* modified Rankin score

^†^Survives FDR correction

### Does the mRS score explain quality of life over the CASE score?

For the total AE sample, hierarchical linear regression analysis found that the CASE score explained a significant proportion of variance for Total NeuroQoL (R² = 0.26, *p* < .001), but the addition of the CASE score did not significantly improve the model (ΔR² = 0.01, *p* = .568). A similar pattern was observed in most NeuroQoL domains, except Fatigue which was not significantly explained by CASE or CASE and mRS. Between 19 and 28% of the variance for Anxiety, Cognitive Function, Positive Affect and Wellbeing, Satisfaction with Social Roles and Activities, Sleep Disturbance and Stigma was explained by the CASE score (Table 4).

## Discussion

Our study aims were twofold. First, this study characterised patient-reported quality of life in patients with AE, an underrepresented population in patient-reported outcome literature. Second, this study investigated whether quality of life is captured by routinely used clinical outcome measures: the mRS and CASE. Our results revealed that a large proportion of AE patients reported poor quality of life despite having been categorised as having a ‘good’ functional score based on mRS and CASE. Additionally, the CASE score was moderately associated with quality of life, such that a higher CASE score was associated with poorer quality of life. Higher mRS scores were also associated with poorer quality of life metrics, but these associations were only found in patients with seropositive AE. Finally, the CASE and mRS did not sufficiently explain quality of life, accounting for only a small proportion of variance. These findings highlight the need for clinicians to utilise patient-reported outcome measures such as the NeuroQoL to assess the impact of AE on their patients’ day-to-day functioning, as clinician-rated outcomes may not accurately reflect their experiences.

In our AE sample, most patients achieved favourable clinical outcomes on the available outcome scales; the median CASE was two, and 76% of the sample had an mRS ≤ 2 at a median of 50 months post-symptom onset. Despite this, a large proportion of patients reported elevated anxiety (48%), cognitive difficulties (66%), fatigue (34%), negative affect and wellbeing (37%), dissatisfaction in social roles and activities (61%), sleep disturbance (43%), and stigma (41%). This reflects previous research; Yokota et al. [25] examined quality of life in 21 patients with AE at a median of 63 months post-disease onset and reported high rates of poor global quality of life (29%), social quality of life (48%), and mental quality of life (29%). Similarly, Blum et al. [26] found poor self-reported psychosocial outcomes in patients recovering from anti-NMDAR antibody-mediated encephalitis at a mean of 4.2 years post-symptom onset. Both aforementioned studies reported that approximately 30% of their samples were unable to return to school/work [25, 26].

Interestingly, our study demonstrated that serostatus influenced quality of life. When compared to the normative data, only the seronegative group had significantly elevated T-scores for Total NeuroQoL. As individuals with seronegative AE often experience greater diagnostic uncertainty, which can lead to delays in diagnosis and access to treatment, this group may be vulnerable to increased psychological distress. Patients with seronegative AE also reported significantly worse cognition than their seropositive peers, which may strongly influence quality of life. These findings highlight the need for more targeted support and clearer clinical communication for patients with seronegative AE.

Preliminary evidence also suggested that premorbid anxiety and/or depression and drug-resistant epilepsy at final follow-up were associated with poor quality of life. Due to relatively small samples, a multiple regression to investigate the predictive value of demographic, treatment, and disease characteristics was not appropriate. Nevertheless, future studies may use our findings to guide hypotheses and explore factors such as steroid dose/duration, use of anti-seizure medication, and other comorbidities (e.g. sleep disturbance).

Our results also demonstrated that poorer quality of life was associated with poorer clinical outcome scores; however, the strength of these relationships was moderate at best and was influenced by serostatus. In the total AE sample, the CASE score was significantly correlated with seven of the eight NeuroQoL scores. However, when the seropositive and seronegative groups were analysed separately, these correlations between CASE score and NeuroQoL scores were more frequent in the seropositive group. Note, however, that not all significant findings survived FDR correction (Supplementary Table 1).

In the current literature, the mRS score is often dichotomised into ‘favourable’ (mRS ≤ 2) and ‘poor’ outcome (mRS > 2). Using this criterion, our study found that Total NeuroQoL, Cognitive Function, Satisfaction in Social Roles and Activities, and Stigma scores were significantly poorer in seropositive AE patients with poor mRS scores compared to those with favourable mRS scores. There were no significant differences in the seronegative group. Our study also repeated these analyses with a stricter definition of ‘favourable’ prognosis of an mRS of 0 or 1. A mRS of 1 is defined as a patient being able to carry out all usual duties and activities despite symptoms, while an mRS of 2 is defined as a patient being unable to perform all previous activities, but able to look after their affairs without assistance. Using this stricter criterion, this study found that seropositive AE patients with mRS scores > 1 had significantly poorer quality of life compared with patients with mRS ≤ 1.

In this study, we also investigated whether using both CASE and the mRS could predict quality of life over utilising just one outcome scale. Results demonstrated that the CASE score and mRS were relatively comparable in capturing quality of life in seropositive AE patients; both scores independently explained between 14 and 50% of variance, with no additional explained variance when taken in conjunction with the other. Results demonstrated that the CASE was better than the mRS at capturing quality of life in seronegative AE patients, but only accounted for 21 to 28% of the variance across four NeuroQoL scores. Note that up to 50% of the variance in quality of life remained unexplained by clinician-rated outcomes. Hence, clinicians should not assume that patients with good functional outcomes are less likely to suffer from psychosocial issues. Rather, they should endeavour to integrate clinical measures and patient-reported outcomes when assessing the actual impact and outcomes after AE.

This study has several limitations. First, despite recruiting across several Australian centres, the study had a relatively small sample size, so comparisons between AE subtypes were not possible, multivariate analyses were underpowered, and the generalisability of the findings are limited. Second, the 6-month window between NeuroQoL and clinical outcome score (CASE and mRS) completion may limit the accuracy of the observed relationships between these outcomes. However, as a majority of our sample are 50-months post-symptom onset and so are in a ‘chronic’ and ‘stable’ phase of their disease, the effect of this temporal window is likely minor. Nevertheless, replication of these findings with concurrently collected measures is required. Third, CASE and mRS scores were retrospectively assigned for patients without these scores on medical records. Fourth, the study design precluded the ability to characterise the evolution of quality of life from the acute to the chronic setting. Future studies should utilise a longitudinal design and examine whether clinical outcome measures in the acute setting are prognostic of long-term quality of life. This study also suffers from selection bias, as the NeuroQoL was provided to patients in a written format, the sample is biased towards those without severe cognitive symptoms who are able to read, understand, and respond to the measure. We attempted to reduce the cognitive burden on patients by selecting fewer measures; however, this meant that other NeuroQoL domains, such as emotional and behavioural dysregulation and physical function could not be explored. Finally, the NeuroQoL does not examine some aspects of quality life, such as sexual function, which limits the comprehensiveness of the reported data.

This study highlighted that poor quality of life is a common occurrence for patients with AE despite recording good outcomes on current clinical assessment. Clinicians should be mindful that clinical outcome scores do not accurately reflect patient experiences, so they should utilise a combination of both clinical and patient-reported outcomes when examining patients’ health. The development of valid and reliable measures for assessing symptom burden in AE is crucial to assess the efficacy of rehabilitation programs, enable more suitable allocation of resources and services, improve patient engagement, and inform healthcare policies.

## Supplementary Information

Below is the link to the electronic supplementary material.

Supplementary Material 1

Supplementary Material 2

Supplementary Material 3

Supplementary Material 4

Supplementary Material 5
