# Supplementary material for: The relationship between patient-reported quality of life and clinician-rated outcome scores in patients with autoimmune encephalitis: a study of the Australian Autoimmune Encephalitis Consortium
Source: Qual Life Res. 2025 Aug 31;34(12):3635–48. doi: 10.1007/s11136-025-04052-4 (PMC12689736; doi:10.1007/s11136-025-04052-4)
Supplement: Supplementary file 2 — Supplementary Material 2 [file 11136_2025_4052_MOESM2_ESM.docx]

**Supplementary Table 2.** Pearson’s correlations between NeuroQoL and demographic/treatment variables for the Total AE sample

|  | **Age at symptom onset (years)** | | **Time to treatment (days)** | | **Time since symptom onset (months)** | |
| --- | --- | --- | --- | --- | --- | --- |
|  | *Pearson’s r* | *p-value* | *Pearson’s r* | *p-value* | *Pearson’s r* | *p-value* |
| **Total** | -0.114 | .418 | 0.212 | .132 | 0.184 | .188 |
| **Anxiety** | -0.090 | .520 | 0.047 | .739 | 0.106 | .444 |
| **Cognitive Function** | -0.011 | .935 | 0.208 | .134 | 0.233 | .090 |
| **Fatigue** | -0.141 | .313 | 0.250 | .074 | 0.182 | .192 |
| **Positive Affect and Wellbeing** | 0.060 | .664 | 0.125 | .373 | -0.056 | .687 |
| **Satisfaction with Social Roles and Activities** | -0.077 | .579 | 0.181 | .194 | 0.153 | .268 |
| **Sleep Disturbance** | -0.262 | .056 | 0.154 | .272 | 0.160 | .248 |
| **Stigma** | 0.003 | .984 | 0.177 | .204 | 0.128 | .356 |
