# Supplementary material for: The relationship between patient-reported quality of life and clinician-rated outcome scores in patients with autoimmune encephalitis: a study of the Australian Autoimmune Encephalitis Consortium
Source: Qual Life Res. 2025 Aug 31;34(12):3635–48. doi: 10.1007/s11136-025-04052-4 (PMC12689736; doi:10.1007/s11136-025-04052-4)
Supplement: Supplementary file 3 — Supplementary Material 3 [file 11136_2025_4052_MOESM3_ESM.docx]

**Supplementary Table 3.** Welch’s t-tests to compare NeuroQoL scores between groups with and without drug resistant epilepsy at final follow up, with and without premorbid anxiety or depression.

| **NeuroQoL Domain** | **Sex** | | ***t*** | **df** | ***p*** | ***d*** |
| --- | --- | --- | --- | --- | --- | --- |
|  | **Female** | **Male** |  |  |  |  |
|  | **Mean (SD)** | **Mean (SD)** |  |  |  |  |
| **Total** | 53.63 (6.48) | 52.55 (7.35) | 0.57 | 50.61 | .574 | 0.16 |
| **Anxiety** | 55.31 (7.43) | 53.46 (8.16) | 0.87 | 51.55 | .388 | 0.24 |
| **Cognitive Function** | 57.56 (9.97) | 59.13 (9.20) | -0.60 | 51.66 | .550 | -0.16 |
| **Fatigue** | 51.76 (10.54) | 48.86 (10.18) | 1.02 | 50.72 | .314 | 0.28 |
| **Positive Affect and Wellbeing** | 50.00 (8.20) | 50.70 (7.12) | -0.33 | 50.98 | .740 | -0.09 |
| **Satisfaction with Social Roles and Activities** | 56.47 (4.70) | 54.33 (5.88) | 1.48 | 49.60 | .145 | 0.40 |
| **Sleep Disturbance** | 54.76 (8.92) | 51.91 (10.58) | 1.07 | 50.55 | .289 | 0.29 |
| **Stigma** | 51.18 (8.69) | 49.47 (8.45) | 0.73 | 51.96 | .466 | 0.20 |

| **NeuroQoL Domain** | **Premorbid anxiety or depression** | | ***t*** | **df** | ***p*** | ***d*** |
| --- | --- | --- | --- | --- | --- | --- |
|  | **No** | **Yes** |  |  |  |  |
|  | **Mean (SD)** | **Mean (SD)** |  |  |  |  |
| **Total** | 51.88 (6.64) | 56.78 (6.55) | -2.33 | 20.64 | .030 | -0.74 |
| **Anxiety** | 52.77 (7.24) | 59.02 (7.67) | -2.67 | 21.67 | .014 | -0.84 |
| **Cognitive Function** | 56.87 (9.72) | 62.55 (7.84) | -2.19 | 28.04 | .037 | -0.64 |
| **Fatigue** | 49.08 (10.43) | 54.00 (9.59) | -1.57 | 22.00 | .130 | -0.49 |
| **Positive Affect and Wellbeing** | 48.92 (7.56) | 54.44 (6.38) | -2.65 | 26.76 | .013**†** | -0.79 |
| **Satisfaction with Social Roles and Activities** | 54.32 (5.36) | 58.48 (4.24) | -2.94 | 28.59 | .006**†** | -0.86 |
| **Sleep Disturbance** | 52.16 (9.23) | 56.69 (10.93) | -1.39 | 19.88 | .181 | -0.45 |
| **Stigma** | 49.03 (8.35) | 54.02 (8.23) | -1.95 | 23.06 | .064 | -0.60 |

| **NeuroQoL Domain** | **Second Line Treatment** | | ***t*** | **df** | ***p*** | ***d*** |
| --- | --- | --- | --- | --- | --- | --- |
|  | **No** | **Yes** |  |  |  |  |
|  | **Mean (SD)** | **Mean (SD)** |  |  |  |  |
| **Total** | 51.66 (7.55) | 53.94 (6.43) | -1.13 | 35.30 | .268 | -0.33 |
| **Anxiety** | 51.68 (8.46) | 56.11 (6.91) | -2.01 | 36.48 | .052 | -0.57 |
| **Cognitive Function** | 57.37 (9.16) | 58.96 (9.85) | -0.61 | 45.02 | .548 | -0.17 |
| **Fatigue** | 48.10 (11.79) | 51.61 (9.33) | -1.13 | 33.31 | .265 | -0.33 |
| **Positive Affect and Wellbeing** | 49.69 (9.64) | 50.78 (6.12) | -0.46 | 30.31 | .647 | -0.14 |
| **Satisfaction with Social Roles and Activities** | 54.61 (6.56) | 55.90 (4.51) | -0.79 | 32.03 | .438 | -0.23 |
| **Sleep Disturbance** | 51.94 (10.29) | 54.23 (9.53) | -0.82 | 40.29 | .417 | -0.23 |
| **Stigma** | 50.85 (7.78) | 49.99 (9.08) | 0.37 | 47.40 | .713 | 0.10 |

| **NeuroQoL Domain** | **DRE at final follow up** | | ***t*** | **df** | ***p*** | ***d*** |
| --- | --- | --- | --- | --- | --- | --- |
|  | **No** | **Yes** |  |  |  |  |
|  | **Mean (SD)** | **Mean (SD)** |  |  |  |  |
| **Total** | 52.05 (6.63) | 58.87 (5.59) | -3.09 | 10.83 | .011**†** | -1.11 |
| **Anxiety** | 53.15 (7.49) | 61.53 (5.47) | -3.76 | 12.11 | .003**†** | -1.28 |
| **Cognitive Function** | 56.79 (9.41) | 67.26 (3.58) | -5.58 | 27.68 | < .001**†** | -1.47 |
| **Fatigue** | 49.69 (10.34) | 53.64 (9.49) | -0.98 | 9.58 | .350 | -0.38 |
| **Positive Affect and Wellbeing** | 50.10 (7.33) | 51.83 (9.49) | -0.49 | 8.52 | .636 | -0.20 |
| **Satisfaction with Social Roles and Activities** | 54.80 (5.44) | 58.83 (3.57) | -2.69 | 13.47 | .018 | -0.87 |
| **Sleep Disturbance** | 51.88 (9.15) | 61.70 (9.72) | -2.66 | 9.29 | .025 | -1.04 |
| **Stigma** | 49.10 (8.46) | 57.33 (4.99) | -3.80 | 15.16 | .002**†** | -1.18 |

*Note.* **†**survive FDR correction
