# Supplementary material for: The relationship between patient-reported quality of life and clinician-rated outcome scores in patients with autoimmune encephalitis: a study of the Australian Autoimmune Encephalitis Consortium
Source: Qual Life Res. 2025 Aug 31;34(12):3635–48. doi: 10.1007/s11136-025-04052-4 (PMC12689736; doi:10.1007/s11136-025-04052-4)
Supplement: Supplementary file 4 — Supplementary Material 4 [file 11136_2025_4052_MOESM4_ESM.docx]

**Supplementary Table 4.** Comparison of NeuroQoL T-scores, mRS and CASE between the seropositive AE sample and seronegative AE sample (Welch’s t-tests).

| **Scale** | **Seropositive AE only**  **Mean (SD)** | **Seronegative AE only**  **Mean (SD)** | ***t*** | **df** | ***p*** | ***d*** |
| --- | --- | --- | --- | --- | --- | --- |
| Total | 51.97 (7.07) | 54.76 (6.40) | 1.49 | 45.87 | .144 | 0.41 |
| Anxiety | 53.88 (7.86) | 55.19 (7.79) | 0.60 | 43.01 | .552 | 0.17 |
| Cognitive Function | 56.22 (10.29) | 61.67 (7.25) | 2.28 | 51.37 | .027 | 0.61 |
| Fatigue | 49.01 (10.34) | 52.23 (10.34) | 1.11 | 42.94 | .272 | 0.31 |
| Positive Affect and Wellbeing | 49.33 (7.91) | 51.96 (7.01) | 1.28 | 46.46 | .207 | 0.35 |
| Satisfaction with Social Roles and Activities | 54.63 (5.78) | 56.61 (4.53) | 1.40 | 49.63 | .167 | 0.38 |
| Sleep Disturbance | 53.29 (9.80) | 53.42 (10.04) | 0.05 | 41.98 | .962 | 0.01 |
| Stigma | 49.09 (7.97) | 52.26 (9.21) | 1.30 | 38.17 | .202 | 0.37 |
| mRS | 1.42 (1.25) | 1.81 (1.12) | 1.18 | 46.08 | .246 | 0.32 |
| CASE | 1.90 (1.92) | 2.75 (3.55) | 0.98 | 26.65 | .336 | 0.30 |

*Note.* Significant difference in Cognitive Function between seropositive and seronegative group did not survive FDR correction (*p*(FDR) = .082).
