# Supplementary material for: The relationship between patient-reported quality of life and clinician-rated outcome scores in patients with autoimmune encephalitis: a study of the Australian Autoimmune Encephalitis Consortium
Source: Qual Life Res. 2025 Aug 31;34(12):3635–48. doi: 10.1007/s11136-025-04052-4 (PMC12689736; doi:10.1007/s11136-025-04052-4)
Supplement: Supplementary file 5 — Supplementary Material 5 [file 11136_2025_4052_MOESM5_ESM.docx]

**Supplementary Table 5.** Frequency of Impairment for the total AE sample, seropositive AE sample, and seronegative sample

| **Scale** | **Seropositive AE only** | | | | **Seronegative AE only** | | | | **Total AE sample**  **(seropositive + seronegative cases)** | | | |
| --- | --- | --- | --- | --- | --- | --- | --- | --- | --- | --- | --- | --- |
|  | **n (%)** | | | | **n (%)** | | | | **n (%)** | | | |
|  | **Normal** | **Mild** | **Moderate** | **Severe** | **Normal** | **Mild** | **Moderate** | **Severe** | **Normal** | **Mild** | **Moderate** | **Normal** |
| Total | 21 (66) | 6 (19) | 5 (16) | 0 (0) | 10 (48) | 5 (24) | 6 (29) | 0 (0) | 31 (58) | 11 (21) | 11 (21) | 0 (0) |
| Anxiety | 18 (55) | 7 (21) | 8 (24) | 0 (0) | 11 (52) | 3 (14) | 7 (33) | 0 (0) | 29 (54) | 10 (19) | 15 (28) | 0 (0) |
| Cognitive Function | 15 (45) | 4 (12) | 13 (40) | 1 (3) | 6 (29) | 3 (14) | 10 (48) | 2 (10) | 21 (39) | 7 (13) | 23 (43) | 3 (6) |
| Fatigue | 23 (72) | 5 (16) | 4 (13) | 0 (0) | 14 (67) | 2 (10) | 5 (24) | 0 (0) | 37 (70) | 7 (13) | 9 (17) | 0 (0) |
| Positive Affect and Wellbeing | 26 (78) | 5 (15) | 2 (6) | 0 (0) | 14 (67) | 3 (14) | 4 (19) | 0 (0) | 40 (74) | 8 (15) | 6 (11) | 0 (0) |
| Satisfaction with Social Roles and Activities | 18 (55) | 9 (27) | 6 (18) | 0 (0) | 7 (33) | 9 (43) | 5 (24) | 0 (0) | 25 (46) | 18 (34) | 11 (20) | 0 (0) |
| Sleep Disturbance | 19 (58) | 6 (18) | 7 (21) | 1 (3) | 13 (62) | 2 (10) | 5 (24) | 1 (5) | 32 (59) | 8 (15) | 12 (22) | 2 (4) |
| Stigma | 24 (73) | 7 (21) | 2 (6) | 0 (0) | 12 (57) | 5 (24) | 4 (19) | 0 (0) | 36 (67) | 16 (22) | 6 (11) | 0 (0) |

Note. Counts and percentages of the AE sample (seropositive cases, seronegative cases, and total sample) reporting normal and poor quality of life. Poor quality of life is categorised based on the severity of symptoms; T-scores less than 55 were classified as “normal”, 55-60 “mild”, 61-70 “moderate”, and greater than 70 “severe”.
